# Comparative Evaluation of MS-based Metabolomics Software and Its Application to Preclinical Alzheimer’s Disease

**DOI:** 10.1038/s41598-018-27031-x

**Published:** 2018-06-18

**Authors:** Ling Hao, Jingxin Wang, David Page, Sanjay Asthana, Henrik Zetterberg, Cynthia Carlsson, Ozioma C. Okonkwo, Lingjun Li

**Affiliations:** 1 School of Pharmacy, University of Wisconsin-Madison, Madison, WI USA; 2 Baylor College of Medicine, Houston, TX USA; 3 Department of Biostatistics & Medical Informatics, University of Wisconsin-Madison, Madison, WI USA; 4 Wisconsin Alzheimer’s Disease Research Center, University of Wisconsin-Madison, Madison, WI USA; 5 Clinical Neurochemistry Laboratory, Sahlgrenska University Hospital Mölndal, Mölndal, Sweden; 6 Institute of Neuroscience and Physiology, Department of Psychiatry and Neurochemistry, the Sahlgrenska Academy at the University of Gothenburg, Mölndal, Sweden; 7 Department of Molecular Neuroscience, UCL Institute of Neurology, London, UK; 8 Dementia Research Institute, London, UK; 9 Department of Chemistry, University of Wisconsin-Madison, Madison, WI USA

## Abstract

Mass spectrometry-based metabolomics has undergone significant progresses in the past decade, with a variety of software packages being developed for data analysis. However, systematic comparison of different metabolomics software tools has rarely been conducted. In this study, several representative software packages were comparatively evaluated throughout the entire pipeline of metabolomics data analysis, including data processing, statistical analysis, feature selection, metabolite identification, pathway analysis, and classification model construction. LC-MS-based metabolomics was applied to preclinical Alzheimer’s disease (AD) using a small cohort of human cerebrospinal fluid (CSF) samples (N = 30). All three software packages, XCMS Online, SIEVE, and Compound Discoverer, provided consistent and reproducible data processing results. A hybrid method combining statistical test and support vector machine feature selection was employed to screen key metabolites, achieving a complementary selection of candidate biomarkers from three software packages. Machine learning classification using candidate biomarkers generated highly accurate and predictive models to classify patients into preclinical AD or control category. Overall, our study demonstrated a systematic evaluation of different MS-based metabolomics software packages for the entire data analysis pipeline which was applied to the candidate biomarker discovery of preclinical AD.

## Introduction

Metabolomics is defined as the systematic study of small molecules profile within a biological system. Characterization of the metabolome offers a wealth of information regarding both enzymatic activities and environmental factors^1,2^. Mass spectrometry (MS) has become a foremost technology for the study of metabolites and their dynamic alterations involved in various diseases^3–6^. In particular, hyphenated MS platforms can provide reproducible detection and sensitive measurements for thousands of metabolites in complex samples by online coupling with separation systems prior to MS analysis, including liquid chromatography (LC), gas chromatography (GC), and capillary electrophoresis (CE)^7–12^. Different MS-based analytical platforms provide complementary coverage of the complex metabolome in a given biological sample. LC-MS does not require chemical derivatization and offers large sample loading capacities for small molecules with high degree of structural diversity. GC-MS is highly reproducible and well suited for thermal stable and volatile compounds or compounds that can be derivatized to be volatile. CE-MS has the ability to separate charged or polar molecules based on charge-to-size ratios with high resolution and relatively small amounts of samples.

As hyphenated MS platforms are widely used for metabolomics analysis as well as the application to disease biomarker discovery, significant challenges have arisen with regard to analyzing the large and multidimensional data sets^13,14^. In terms of studies for human diseases, sample size and amount are often very limited with the number of compounds far exceeding the number of human subjects. Therefore, sophisticated bioinformatics tools and data-mining technologies are required for automated data analysis and evaluation. With a focus on LC-MS and CE-MS-based metabolomics, a number of freely available and commercial software packages have been developed for data analysis, such as XCMS^15–17^, MZmine^18^, MetAlign^19^, MAVEN^20^, SIEVE (Thermo), Progenesis QI (Waters), MetaboScape (Brucker), and most recently, Compound Discoverer (Thermo). Each software package possesses unique advantages in different steps of pre-processing, data analysis, visualization, and interpretation^21,22^. However, systematic comparison of different software packages has rarely been conducted.

In this study, representative software packages (XCMS Online 3.5.1, SIEVE 2.2, and Compound Discoverer 2.0) were selected and comparatively evaluated throughout the entire pipeline of metabolomics data analysis, including data processing, statistical analysis, feature selection, metabolite identification, pathway analysis, and classification model construction. The metabolomics analysis was performed using a small cohort of human cerebrospinal fluid (CSF) samples (N = 30) and further applied to the preclinical Alzheimer’s disease (AD).

AD is a neurodegenerative disorder affecting millions of elderly people worldwide. AD patients suffer from progressive syndromes of memory loss, language impairment, and behavioral disturbance. Pathophysiologically, AD is characterized by the presence of extracellular amyloid β (Aβ) plaque and intracellular neurofibrillary aggregates of protein tau in the brain^23^. The concept of AD has evolved to be a continuum over the past decade, with a new disease framework including the preclinical (pre-symptomatic) stage, mild cognitive impairment, and dementia^24^. The pathophysiological process of AD starts years before the emergence of clinical syndrome, yet unequivocal diagnosis and treatment in the early phase of AD is still lacking. It is highly possible that patients could be optimally treated in the preclinical stage of AD before the occurrence of clinical symptoms. CSF circulates within the brain ventricular system, maintains metabolic homeostasis of the brain, and is therefore the most direct and valuable biofluid sample to evaluate the dysfunction of the central nervous system. Discovering the metabolic changes in CSF samples derived from people in the preclinical stage of AD can provide critical insights into disease progression and support the early diagnosis and treatment of AD^25–27^.

## Results

### Data processing evaluations

**Figure 1:**
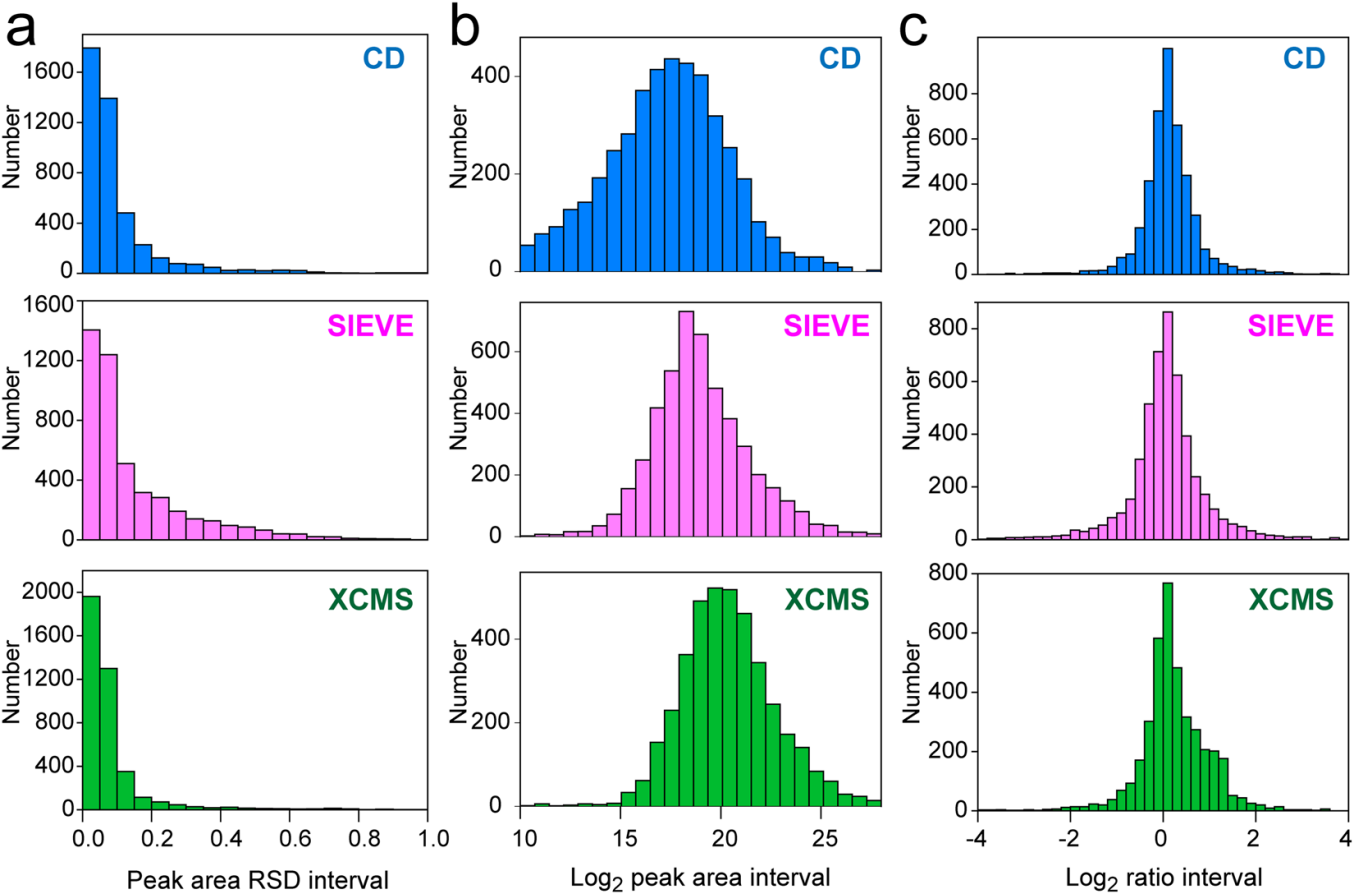
Data processing evaluation of three software packages, Compound Discoverer, SIEVE, and XCMS Online. (**a**) Histograms of peak area relative standard deviations (RSD) of all detected features in QC data set. (**b**) Histograms of log_2_-transformed peak areas of all detected features in QC data set. (**c**) Histograms of log_2_-transformed ratios of all detected features in preclinical AD vs. control data set.

Efficient and reliable data processing is the first key step toward successful data analysis and biologically important findings. Herein, we assessed the data processing performance of three widely-used software packages, XCMS Online, SIEVE 2.2, and Compound Discoverer 2.0. Firstly, a pooled mixture of aliquots from all CSF metabolite specimens was prepared as a quality control (QC) sample. QC injections (n = 3) were used to evaluate the consistency, reproducibility, and dynamic range of data processing. Ideally, the results of technical replicates should be perfectly repetitive with zero deviation. But inevitable variations can be introduced from both instrument platform and data analysis platform. Since our previous metabolomics study has demonstrated the excellent reproducibility of the current LC-MS/MS platform^4^, data analysis consistency of three software packages can be compared using the peak area relative standard deviation (RSD) of all the compounds detected from QC injections. More than 4000 compounds (combining positive and negative electrospray ionization modes, deisotoped and de-adducted) were detected in the QC sample. Histograms in Fig. 1a indicate excellent consistency and reproducibility of all three software packages. Over 60% of the detected compounds obtained peak area RSDs lower than 0.1. XCMS Online software yielded the most reproducibly integrated peak areas with nearly 2000 compounds’ RSD lower than 0.05. The consistency of peak extraction and integration is essential as subsequent statistical analyses are conducted based on the values of peak areas. The different variabilities of peak areas among three software packages could be caused by the intrinsic algorithms used for peak detection and integration and could also be influenced by chromatography alignment^21^.

One of the key challenges of metabolomics profiling is the high dynamic range of metabolite concentrations present in biological samples. Different classes of metabolites often present distinct LC affinities and ionization efficiencies on LC-ESI-MS platform. The collective effects of large concentration range and structural diversity result in the high peak area dynamic range of LC-MS-based metabolomics datasets, which was found to be 6 orders of magnitude. The histograms of log_2_-transformed peak areas from all detected compounds showed approximately normal distribution, where majority of the detected peaks were within the range of 1e^5^ to 5e^6^ peak area (Fig. 1b). However, the mean of the histogram distribution followed the trend of CD < SIEVE < XCMS Online, and CD software also generated more left-skewed distribution, suggesting that CD software extracted the largest number of low-abundance metabolites. Furthermore, only CD software can extract both ESI + and ESI- spectra simultaneously from data files in a single analysis.

### Statistical evaluations

In the preclinical AD vs. control dataset, the total numbers of detected compounds (ESI+ and ESI−) were 4389, 4778, and 4004 using CD, SIEVE, and XCMS Online respectively. Both univariate and multivariate statistical analyses were conducted for the comparative assessment of the three software packages. In the univariate statistical test, the discrimination of individual metabolites between preclinical AD and control groups were represented by *t-*test *p-*values and ratios. *P*-values need to be corrected for multiple hypotheses testing to limit the number of false positives and account for the possible interactions among detected compounds. Both CD and XCMS Online software provide adjusted *p*-value for each quantified mass feature. As illustrated in the histograms in Fig. 1c, ratios from all three software packages followed normal distribution, where the majority of the compounds remained unchanged between preclinical AD vs. control groups. It is worth mentioning that XCMS Online offers more choices of statistical tests than CD and SIEVE software for two-group or multiple group comparisons, such as paired t-test, Mann-Whitney test, Wilcoxon signed-rank test, and ANOVA^17^.

**Figure 2:**
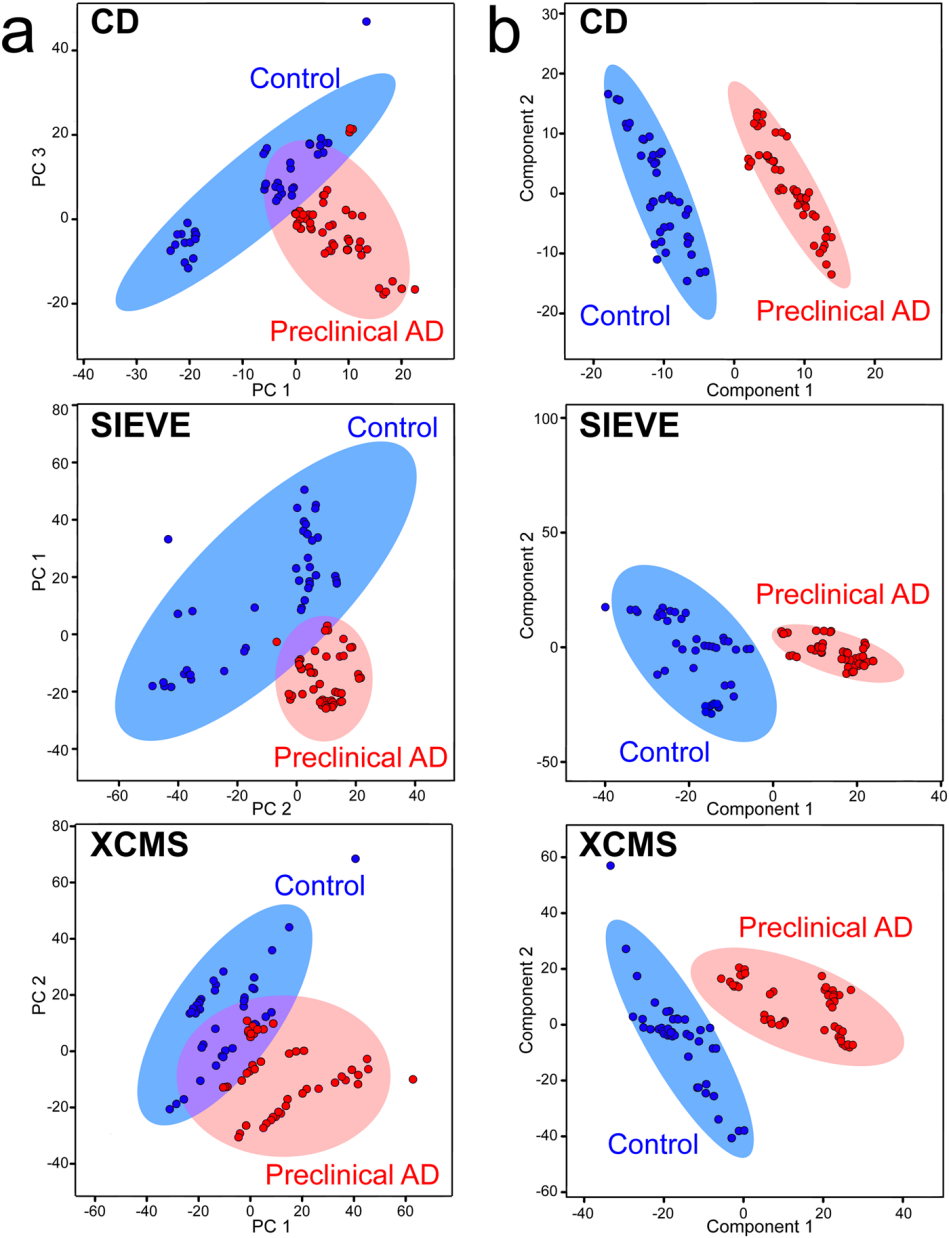
Multivariate statistical analyses of human CSF samples in preclinical AD vs. control vs. QC groups. (**a**) Principal component analysis. (**b**) Partial least squares-discriminant analysis.

For multivariate analysis, PCA is conducted first to visualize metabolite fingerprints in a reduced dimensional subspace. None of the software packages yielded complete separation of preclinical AD and control groups in PCA plots (Fig. 2a). PLS-DA analysis was then carried out to sharpen the separation among groups. Complete separation among preclinical AD, control and QC groups were achieved with all three software packages with PLS-DA analysis (Fig. 2b). Compound discoverer software presented the most distinct clustering and separation among different groups. The cross-validated accuracies of the established PLS-DA models were 0.97, 0.96, and 0.93 for CD, SIEVE, and XCMS Online, respectively.

### Application to biomarker discovery of preclinical AD

**Figure 3:**
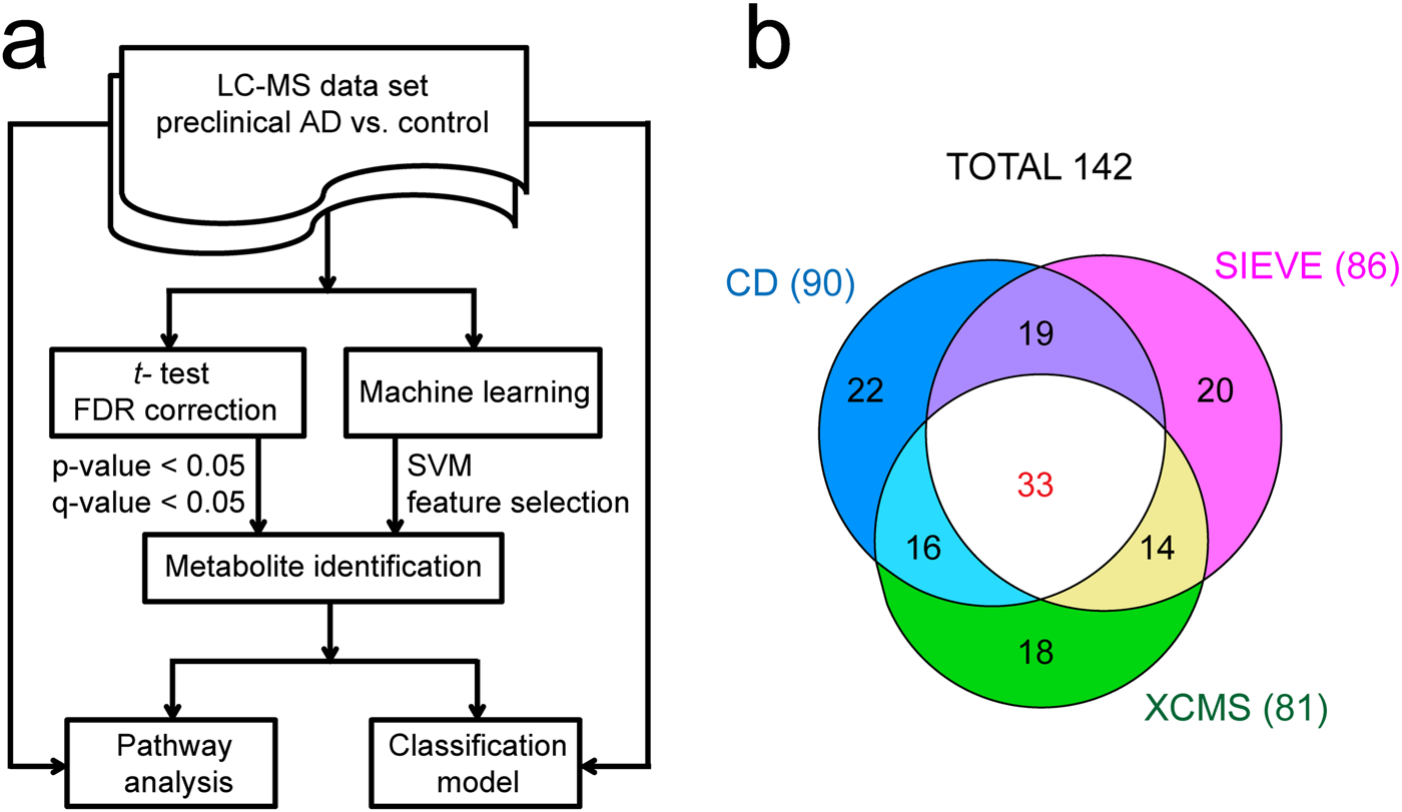
Data analysis flowchart (**a**) and the overlapping candidate biomarkers of preclinical AD resulted from three software packages (**b**).

**Figure 4:**
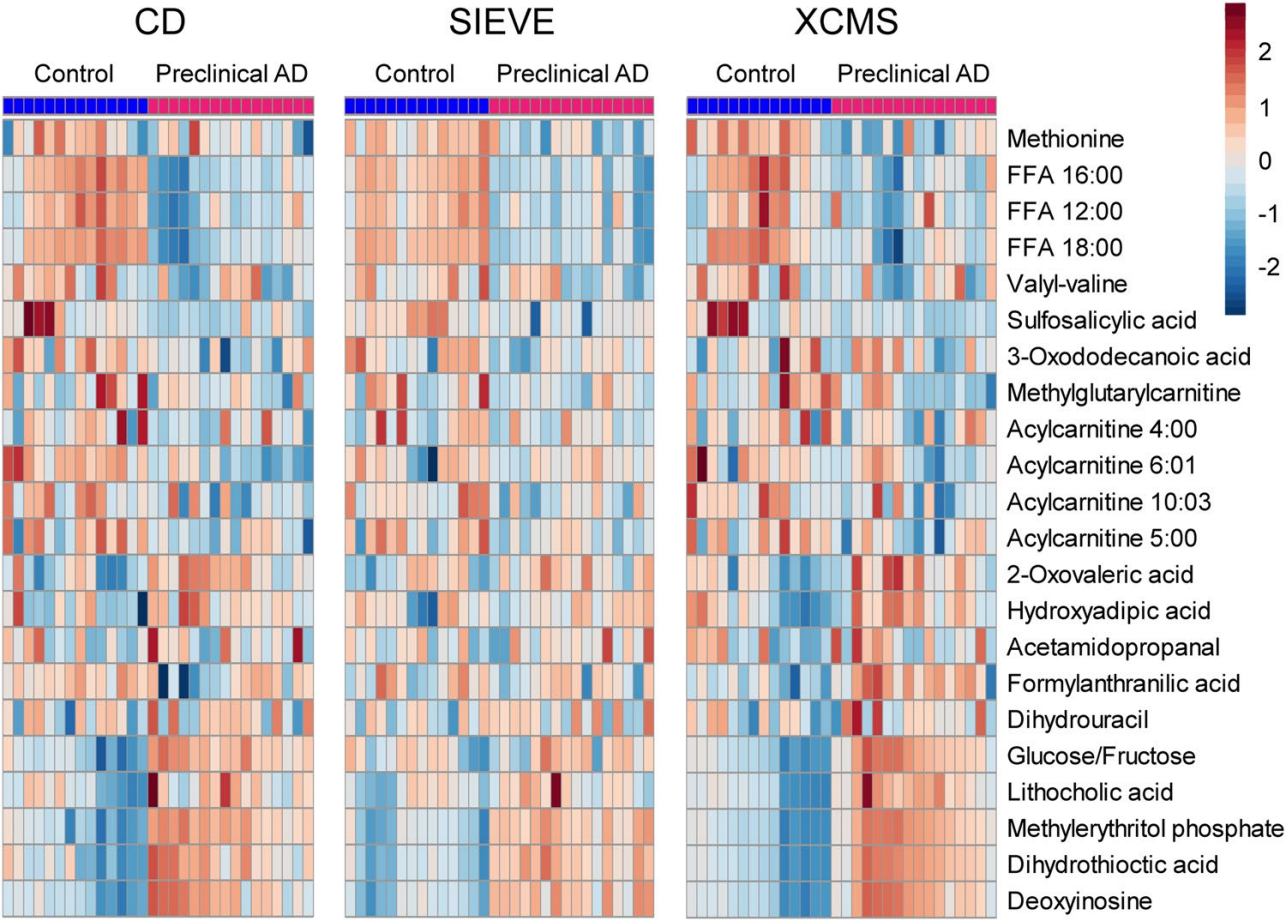
Heatmaps of representative candidate biomarkers among shared metabolites from three software packages. The data was log transformed and auto-scaled.

In order to select a panel of most significantly altered features as candidate biomarkers, we have designed a hybrid method combining traditional statistical tests and machine learning feature selection as illustrated in Fig. 3a. The process of biomarker selection and metabolite identification resulted 90, 86, and 81 candidate metabolite biomarkers from CD, SIEVE, and XCMS Online, respectively. As shown in Fig. 3b, the three software packages provided complementary coverage of candidate biomarkers with over 75% shared metabolites between at least two platforms. All 142 metabolites were identified in three software packages, but some of them did not reach statistical significance in one or two software due to the different results of peak integration generated from different software packages. The complete peak list of these 142 metabolites is provided in Supplemental Table S1. The heatmaps of representative metabolite biomarkers (fold change >1.2 among shared metabolites) are displayed in Fig. 4. Each shade of color represents the relative expression level of certain metabolite in an individual human CSF sample. The overall trends of color distributions are similar among the three software packages, indicating consistent and effective analyses of the CSF data set by all three packages.

Candidate metabolite biomarkers generated through three software packages were further evaluated by building binary classification models to differentiate preclinical AD and control patients. Ideally for datasets with a large number of samples, classification models can be constructed on a training set and tested on an independent test set to avoid biased estimates of classifier accuracy. However, for many omics studies, clinical human samples are often very limited, especially for precious samples like human CSF. Leave-one-patient-out cross-validation was therefore used in this study for an estimate of the binary classification model accuracy. The SVM algorithm was selected for machine learning classification, which has gained great success in analyzing gene expression data and handling noisy data in omics studies^4,28–31^. In order to minimize the over-fitting of the classification model, the process of biomarker selection was repeated on every fold of the cross-validation to generate the cross-validated receiver operating characteristic (ROC) curve and its area under the curve (AUC) value^4^. As compared in Table 1, all three software packages achieved highly accurate classification results with sensitivity, specificity, precision and AUC ROC greater than 0.93. If the established model can be validated in the future with a larger sample size (N > 100), it will possess great potential to be used for developing objective diagnostic test of preclinical AD via CSF sampling.

**Table 1:** Binary classification performance of candidate biomarkers to differentiate preclinical AD and control groups using three software packages.

Software	Feature number	Sensitivity	Specificity	Precision	ROC area
CD	90	0.967	0.962	0.969	0.964
SIEVE	86	0.967	0.962	0.969	0.964
XCMS Online	81	0.933	0.933	0.933	0.933

**Figure 5:**
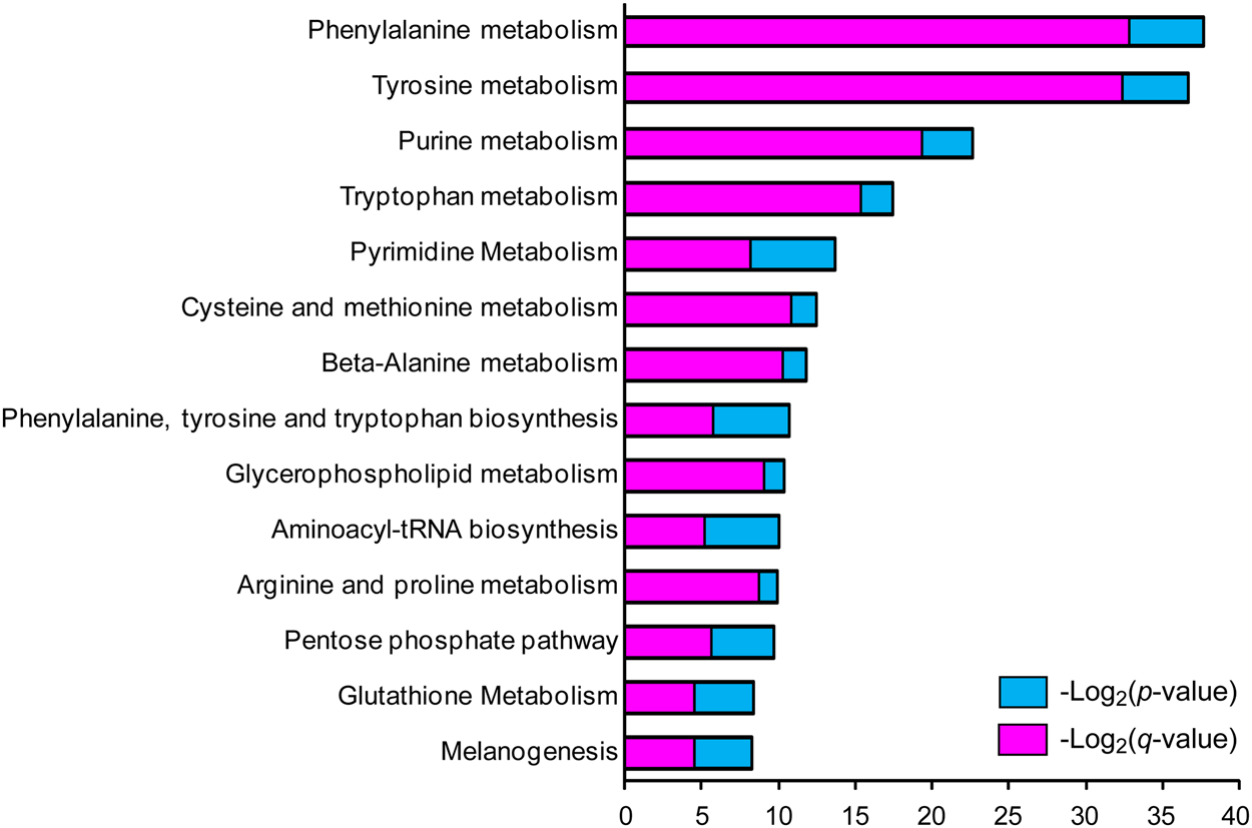
Dysregulated Metabolic pathways in human CSF of preclinical AD vs. control patients. *P*-values and corrected *p*-values of metabolic pathways were calculated by weighing the number of compounds in the set against in the background in MBROLE software.

Functional enrichment of the selected 142 metabolites was performed with MBROLE 2.0 tool based on metabolites’ categorical annotations in multiple databases^32^. Categorical annotations include metabolic pathways, biofunctions, chemical classifications, and possible association with diseases. HMDB and LIPID MAPS taxonomies indicated that the most enriched chemical groups of candidate biomarkers were carboxylic acids, amino acids, fatty acyls, fatty acids and conjugates, pyrimidines, and nucleosides and analogues. The most enriched cellular locations of candidate biomarkers were cytoplasm, nucleus, mitochondria, endoplasmic reticulum, lysosome, golgi apparatus, peroxisome, and membrane. Six metabolites were directly assigned to AD classification in HMDB based on literature record, including glutamate, tryptophan, glucose, tyrosine, glycerophosphocholine, and homocitric acids. Metabolic pathway analysis was conducted in both MBROLE and Compound Discoverer software through KEGG database. *P*-values and FDR corrected *p*-value of metabolic pathways were calculated by weighing the number of compounds in the set against in the background in MBROLE^32^. Fourteen metabolic pathways were significantly dysregulated in preclinical AD patient vs. control groups (Fig. 5).

## Discussion

In this study, different MS-based metabolomics software platforms were compared and evaluated throughout the entire data analysis workflow and disease biomarker discovery. Recognizing a variety of open-source or commercial software available for metabolomics data analysis, we selected three representative and widely-used software packages, XCMS Online 3.5.1, SIEVE^TM^ 2.2, and Compound Discoverer^TM^ 2.0. Each software package possesses unique characteristics for data analysis. For instance, XCMS online offers more options for statistical tests than other software but normalization function is not available during data processing. SIEVE presents separate view of peak alignment (both chromatography and alignment scores) allowing optima peak alignment to be performed before feature detection but has no MS/MS matching function for metabolite identification. Compound Discoverer is most recently developed and able to simultaneously process polarity switching data format, yet the comprehensive user-defined workflow requires sufficient knowledge of each node for the best design. In addition, XCMS online requires data upload to the website which could take a couple hours, while SEIVE and CD are installed locally but have system requirements (e.g. Memory, processor) for the local computer to process large scale omics data. XCMS online uses METLIN^33^ database for metabolite identification (MS and MS/MS level), SIEVE and CD use ChemSpider for accurate mass matching, and CD can also search MS and MS/MS data against mzCloud database (https://www.mzcloud.org). We recognize that software packages undergo continuous update with new versions and it is also unrealistic to perform data analysis using multiple software platform in the routine metabolomics analysis, our study offers informative reference and starting point for selecting the most appropriate software platforms based on specific needs for metabolomics data analysis.

LC-MS-based human CSF metabolomics analysis was applied to the candidate biomarker discovery of preclinical AD using a small cohort of patient samples (14 Control and 16 Preclinical). three software packages provided complementary selection of candidate biomarkers and were highly predictive to classify patients into diseased or control groups. Ideally, the resulted candidate biomarkers should be independent on the software used for data analysis, but due the intrinsic differences of algorithms in these software packages and the data processing variations, the results (the total of 142 metabolites) shared about 75% candidate biomarkers between two software platforms. As inter-laboratory reproducibility is one of the major challenges in the omics field, data analysis variations due to different software used across labs must be carefully considered. Using multiple metabolomics software packages for data processing and only considering the overlapping features for subsequent analysis could be beneficial to rule out false positive features, and more likely lead to the discovery of more robust biomarkers. This aspect should be taken into consideration for studies aimed at biomarker discovery. Proper S/N cutoff thresholds should also be carefully determined to ensure the best data quality for metabolomics studies.

Many dysregulated metabolites and metabolic pathways have been reported to potentially correlate with neurodegenerative diseases; particularly their functional roles involved in neurotransmission, oxidative stress, and neuroinflammation^34–36^. Tryptophan metabolism pathway was altered in CSF of preclinical AD patients with a total of 29 identified metabolites. The major route of tryptophan metabolism, kynurenine pathway, is a key regulator of both neuroprotective and neurotoxic compounds and has found to be disturbed in neurological diseases including AD^37^. Tryptophan, kynurenine, and kynurenic acid were all decreased in CSF of preclinical AD patients, which was in agreement with another study suggested the correlation between cognitive function and kynurenine pathway metabolites^26,38–40^. Tryptophan also plays fundamental roles in the synthesis of neurotransmitters like serotonin, melatonin, and tryptamine. Ample evidences indicated that cognitive and memory impairment in early stage of AD patients begins with the inefficiency of hippocampal synaptic functions involved in neurotransmitter systems^41^. Both serotonin and its major metabolite 5-hydroxyindoleacetate were significantly decreased in CSF of preclinical AD patients. In consistent with the dysfunction of glutamatergic synapse reported in early stage of AD^41^, both glutamate and glutamine were significantly changed and selected as candidate biomarkers in CSF of preclinical AD patients. Moreover, aminoacyl-tRNAs are important translation substrates for protein synthesis, and aminoacyl-tRNA synthetases (AARS) participate in the biosynthesis of signaling molecules, dinucleotide polyphosphates, which can stimulate GABA release in CNS systems^42^. A member of AARS, TrypRS, has also been reported to be a potential marker for AD pathology in a transgenic mouse model of the disease^43^. The dysregulation of AARS pathway agrees well with another plasma and CSF metabolomics study of AD^26^. Higher level of homocysteine, caused by the deficiencies in homocysteine re-methylation cofactors vitamin B12 and B9 (folate), has found to be directly associated with decreased cognitive performance in the elderly^44^, which is consistent with our results. In addition, glutathione metabolism was found to be disturbed in preclinical AD which contributes to oxidative stress related to AD^45^. The increased oxidative stress during AD progression may also contribute to neuroinflammation, mitochondrial dysfunction, and synaptic dysfunction^34^. Glycerophospholid-derived lipid mediators play important roles in these bioprocesses and were found to be disturbed in the present study^36,46^. Although exact mechanisms underlying these disturbed metabolic pathways in AD are still unclear, the identified key metabolites and pathways provide key molecular targets for future mechanistic studies.

## Methods

### Participants

Thirty enrollees in the Wisconsin Alzheimer’s Disease Research Center (WADRC) participated in this study. They comprised sixteen Stage 3 preclinical AD individuals and fourteen age-matched control individuals who were not in the AD pathway. Control subjects were cognitively normal individuals determined by comprehensive neuropsychological assessments and enrolled in the WADRC without family history of AD. Detailed inclusion/exclusion criteria for patient recruitment are described elsewhere^24,47^. The average age of the total sample was 61 ± 6 years. The University of Wisconsin Institutional Review Board (IRB) approved all study procedures, and each enrollee provided a signed informed consent form before participation. All methods were performed in accordance with the IRB guideline and regulation. The clinical information of recruited human subjects is provided in Supplemental Table S2.

### CSF sample collection and preparation

Human CSF samples were collected by lumbar puncture at L3/4 or L4/5 following local anesthesia in the morning after 12-hour fast. Each CSF sample was collected via a syringe into a sample collection tube, gently mixed to avoid gradient effect, and centrifuged at 2000 g for 10 min. The supernatant was collected, sub-aliquoted, and stored at −80 °C until use. One milliliter of each CSF sample was thawed on ice and metabolite extraction was achieved by using 3 kDa molecular weight cut-off (MWCO) ultracentrifugation (Millipore Amicon Ultra, MA). The flow-through fraction was collected as CSF metabolite fraction.

### LC-MS/MS analysis

Human CSF metabolite samples were analyzed using a Dionex UltiMate 3000 LC system coupled with a Q-Exactive^TM^ Orbitrap mass spectrometer (San Jose, CA), operated on both positive and negative ESI mode. Mobile phase A was 0.1% formic acid in water and mobile phase B was 0.1% formic acid in methanol. Metabolites were separated on a biphenyl column (Phenomenex, 75.1 μm × 150 mm, 1.7 μm, 100 Å) at an LC flow rate of 0.3 ml/min and a column temperature of 35 °C. The 15 min gradient for positive ESI mode was set as follows: 0–5 min, 0–2% solvent B; 5–10 min, 2–50% solvent B; 10–11 min, 50–90% solvent B; 11–13 min, 90% solvent B; 13–15 min, 0% solvent B. The 11 min gradient for negative ESI mode was set as follows: 0–4 min, 0–2% solvent B; 4–7 min, 2–90% solvent B; 7–9 min, 90% solvent B; 9–11 min, 0% solvent B. The injection volume was 5 μL, and each sample was injected in triplicates. Injection order was randomized, and the group information was blinded for LC-MS analysis. Full MS scans were acquired from *m/z* 70–1000 at a resolution of 70 K. Automatic gain control (AGC) target was 1 × 10^6^ and maximum injection time (max IT) was 100 ms. The targeted LC-MS/MS experiments were conducted with an inclusion list of accurate masses and retention times for the purpose of metabolite identification. Resolution was 17.5 K, AGC target was 5 × 10^5^, max IT was 50 ms, isolation window was 1 *m/z*, and normalized collision energy was 30% with higher-energy collisional dissociation fragmentation. LC-MS instrument was controlled by Thermo Scientific Xcalibur 2.2 software.

### Data processing by three software packages

Raw data files were independently processed by SIEVE^TM^ 2.2, XCMS Online 3.5.1, and Compound Discoverer^TM^ 2.0 software for metabolomics data analysis. A blank sample was used for background subtraction and noise removal during the pre-processing step.

Commercial SIEVE software was developed by Thermo Scientific as a differential analysis software for both label-free metabolomics and proteomics data analyses. Raw metabolomics data files of preclinical AD, control, and QC groups were processed by SIEVE^TM^ 2.2 with peak alignment and framing algorithm and the experimental type of Control Compare Trend. A QC data file was used as the reference file for peak alignment. The frame time width was 2 min and *m/z* width was 5 ppm. The intensity threshold for component extraction was 1e^6^, and signal-to-noise ratio was 3. ICIS algorithm was used for peak detection. The maximum retention time shift for peak alignment was 0.2 min. Total ion current (TIC) normalization embedded in SIEVE was conducted to reduce instrumental variation before statistical analysis.

XCMS Online is a freely available platform, developed by the Scripps Center for untargeted metabolomics data analysis (http://xcmsonline.scripps.edu). Raw datasets were converted to.mzXML format using the Proteowizard MSConvert tool, and uploaded to XCMS Online for data processing. (Note that the current version of XCMS Online also accept raw data files from several vendors) Multigroup analysis job was created for preclinical AD, control, and QC groups. The predefined parameter set for orbitrap instrument was used and adjusted to be similar as the SIEVE parameters listed above, if possible. CentWave algorithm was used for feature detection and orbitrap settings for retention-time correction. Since normalization function is not available in XCMS Online, normalization to the sum is performed in an Excel file after data processing.

Compound Discoverer (CD) is a relatively new software released by Thermo Scientific this year for targeted and untargeted metabolomics data analysis. Custom designed workflow was established for spectra alignment, compound detection, grouping, metabolite identification, and pathway analysis. The detailed workflow is provided in Supplemental Figure S1. Chromatographic alignment, compound detection, and accurate mass identification parameters were set as the same with SIEVE. Compound peak areas were normalized to the constant sum using embedded function before statistical analysis.

### Statistical analysis

Data processing of each software package yielded a multi-dimensional peak table including accurate *m/z*, molecular weight, retention time, compound formula, peak area and other statistical information. Positive and negative ESI data were separately processed and peak tables were combined into an Excel file. Features with >50% missing values were removed. Compound peak areas were log_2_-transformed for subsequent statistical analyses.

Univariate Student’s *t-*test was then performed to generate a *p*-value for each detected compound, together with an average fold change, between preclinical AD and control groups, together with an average fold change. The *p*-values were ranked to calculate FDR corrected *p*-value by the Benjamini-Hochberg procedure as implemented in R package. The threshold of statistical significance was set at both *p*-value and corrected *p*-value lower than 0.05. Multivariate statistical analyses, principal component analysis (PCA) and partial least squares-discriminant analysis (PLS-DA), were also carried out using the web-based software MetaboAnalyst 3.0 after log transformation and auto-scaling^48^.

### Metabolite identification and pathway analysis

Metabolite identification was achieved by accurate mass matching, MS/MS matching, and standard confirmation, following the designed flowchart as described previously^4^. All three software packages provide accurate mass matching functions for tentative metabolite identification, where *m/z* tolerance was set at 10 ppm. Kyoto Encyclopedia of Genes and Genomes (KEGG)^49^, Human Metabolome Database (HMDB)^50^, Madison Metabolomics Consortium Database (MMCD)^51^, and LIPID MAPS^52^ were selected for database searching through ChemSpider for both CD and SIEVE software. Metabolite identification for XCMS Online was achieved by searching against METLIN^33^ metabolite database. An in-house lipid library with more than 2000 entries was used to assist with lipid identification with an accurate mass tolerance of 10 ppm. For MS/MS matching, XCMS Online and CD software have the capability to search MS/MS data against METLIN and mzCloud database, respectively. Additionally, LC-MS/MS spectra were searched against a web-based MetFrag software to provide complementary coverage^53^. Metabolite IDs were also confirmed with an in-house metabolite library and available metabolite standards by comparing their LC retention time, precursor m/z and MS/MS spectra. Metabolic pathway analysis function is available in the CD software where metabolite IDs were mapped into the KEGG pathways. The current version of XCMS Online also support pathway analysis and visualization through the new Pathway Cloud Plot. The pathway function in SIEVE can be optionally licensed but was not purchased in the present study.

### Machine learning feature selection and classification

Machine learning feature selection was achieved by WEKA 3.8 software^54^, specifically employing the support vector machine algorithm to evaluate features’ contributions to the task of separating preclinical AD vs. control groups. Chromatographic peak areas of detected compounds were averaged across technical replicates and input into WEKA. We ran the SVM with a linear kernel, which maps the original feature space into a high dimensional feature space capturing feature interactions in additional to the original features. We employed our previously described method^4^ combining SVM feature selection and traditional statistical test for feature selection. Briefly, all input features were ranked based on their contributions to separate two groups and the top 200 ranked features were then overlapped with statistically significant features (both *p*-value and corrected *p*-value lower than 0.05) for subsequent metabolite identification. The process of biomarker selection was conducted independently for the data set generated from each software package.

For machine learning classification, the peak table containing peak areas of identified candidate biomarkers from each software package was directed into WEKA software. Binary classification model was constructed by leave-one-patient-out cross-validation via linear SVM algorithm. Thirty folds of cross-validation were carried out, where each fold involves withholding one patient as a test set and the rest of 29 patients as a training set. The sensitivity, specificity, and precision of the predictive model were evaluated for classifying patients into preclinical AD or control group.

Supporting data is available. Supplementary data includes: the complete list of 142 identified dysregulated metabolites of preclinical AD from three software packages, clinical information of recruited human subjects, and custom workflow in Compound Discoverer software. Raw LC-MS/MS data are available upon request.

## Electronic supplementary material

Supplementary Information
