# Supplementary material for: Comparative Evaluation of MS-based Metabolomics Software and Its Application to Preclinical Alzheimer’s Disease
Source: Sci Rep. 2018 Jun 18;8:9291. doi: 10.1038/s41598-018-27031-x (PMC6006240; doi:10.1038/s41598-018-27031-x)
Supplement: Supplementary file 1 — Supplementary Information [file 41598_2018_27031_MOESM1_ESM.docx]

**Comparative Evaluation of MS-based Metabolomics Software and Its Application to Preclinical Alzheimer's Disease**

Ling Hao^1^, Jingxin Wang^2^, David Page^3^, Sanjay Asthana^4^, Henrik Zetterberg^5,6,7,8^, Cynthia Carlsson^4^, Ozioma Okonkwo^4^, and Lingjun Li^1,9*^

^1^School of Pharmacy, University of Wisconsin-Madison, Madison, WI, USA; ^2^Baylor College of Medicine, Houston, TX, USA; ^3^Department of Biostatistics & Medical Informatics, University of Wisconsin-Madison, Madison, WI, USA; ^4^Wisconsin Alzheimer's Disease Research Center, University of Wisconsin-Madison, Madison, WI, USA; ^5^Clinical Neurochemistry Laboratory, Sahlgrenska University Hospital Mölndal, Sweden; ^6^Institute of Neuroscience and Physiology, Department of Psychiatry and Neurochemistry, the Sahlgrenska Academy at the University of Gothenburg, Mölndal, Sweden; ^7^Department of Molecular Neuroscience, UCL Institute of Neurology, London, UK; ^8^UK Dementia Research Institute, London, UK; ^9^Department of Chemistry, University of Wisconsin-Madison, Madison, WI, USA.

*Corresponding author

Tel.: +1 (608) 265-8491

Fax: +1 (608) 262-5345

E-mail: [lingjun.li@wisc.edu](mailto:lingjun.li@wisc.edu)

**Table of Contents**

**Supplemental Table S1**. The complete list of 142 identified dysregulated metabolites in preclinical AD vs. control group from three software packages.

**Supplemental Table S2**. Clinical information of recruited human subjects.

**Supplemental Figure S1**. Custom workflow in Compound Discoverer software.

**Supplemental Table S1**. The complete list of 142 identified candidate metabolite biomarkers of preclinical AD from three software packages.

| Name | ESI | detected MW | △ppm | RT(min) | Ratio | *p*-value | Corrected *p*-value | Software |
| --- | --- | --- | --- | --- | --- | --- | --- | --- |
| 3-Oxododecanoic acid | ESI- | 214.1570 | 0.4 | 10.6 | 0.8 | 2.9E-29 | 1.6E-26 | Shared |
| 3-Hydroxy-  dodecanedioic acid | ESI- | 246.1469 | 0.6 | 7.2 | 0.9 | 2.0E-25 | 1.2E-22 | Shared |
| Undecanoylglycine | ESI- | 243.1836 | 0.7 | 7.5 | 0.9 | 6.7E-24 | 8.3E-22 | Shared |
| Methylerythritol phosphate | ESI- | 216.0399 | 0.1 | 0.9 | 2.3 | 1.7E-21 | 8.9E-20 | Shared |
| Glutamine | ESI+ - | 146.0681 | 7.2 | 0.8 | 1.1 | 3.0E-21 | 1.1E-19 | Shared |
| FFA 18:0 | ESI- | 284.2721 | 2.0 | 8.5 | 0.5 | 9.4E-20 | 3.1E-18 | Shared |
| Glucose/Fructose | ESI- | 180.0628 | 3.3 | 0.8 | 2.0 | 1.6E-19 | 8.8E-18 | Shared |
| FAHFA 22:1 | ESI- | 368.2931 | 1.1 | 8.5 | 0.9 | 2.7E-17 | 4.9E-16 | Shared |
| Dihydrothioctic acid | ESI- | 208.0580 | 5.9 | 0.8 | 3.7 | 4.8E-16 | 6.4E-15 | Shared |
| Oxohexadecanoic acid | ESI- | 270.2198 | 1.1 | 8.1 | 0.9 | 1.9E-15 | 2.8E-11 | Shared |
| Deoxyinosine | ESI- | 252.0848 | 4.3 | 0.9 | 5.0 | 4.3E-15 | 4.3E-14 | Shared |
| FFA 16:0 | ESI- | 256.2406 | 1.3 | 8.3 | 0.4 | 4.6E-15 | 4.6E-14 | Shared |
| Succinyl-glutamate  5-semialdehyde | ESI- | 231.0742 | 0.5 | 1.3 | 0.9 | 4.1E-13 | 2.5E-12 | Shared |
| Sulfosalicylic acid | ESI+ | 217.9875 | 4.4 | 2.9 | 0.4 | 4.7E-12 | 2.2E-11 | Shared |
| Uridine | ESI- | 244.0698 | 1.0 | 1.4 | 0.9 | 6.1E-12 | 3.1E-09 | Shared |
| 3-Dehydroquinic acid | ESI- | 190.0472 | 2.7 | 1.1 | 0.9 | 2.1E-11 | 8.8E-11 | Shared |
| Pimelic acid | ESI- | 160.0729 | 3.9 | 6.2 | 1.2 | 4.7E-11 | 1.3E-10 | Shared |
| Uric acid | ESI- | 168.0279 | 2.4 | 1.0 | 0.9 | 5.8E-11 | 2.2E-10 | Shared |
| Formylanthranilic acid | ESI- | 165.0419 | 4.3 | 4.7 | 1.6 | 1.0E-10 | 2.7E-10 | Shared |
| FFA 12:0 | ESI- | 200.1773 | 1.8 | 8.0 | 0.4 | 1.1E-10 | 4.0E-10 | Shared |
| Methionine | ESI+ - | 149.0504 | 4.2 | 1.2 | 0.8 | 2.7E-09 | 6.1E-09 | Shared |
| Tyrosine | ESI+ - | 181.0731 | 4.6 | 1.3 | 1.1 | 4.3E-09 | 9.4E-09 | Shared |
| Acylcarnitine 6:1 | ESI+ - | 257.1620 | 2.7 | 11.7 | 0.8 | 8.5E-09 | 1.7E-04 | Shared |
| Acylcarnitine 5:0 | ESI+ | 245.1620 | 2.9 | 8.8 | 0.8 | 6.0E-06 | 4.0E-05 | Shared |
| 2-Oxovaleric acid | ESI+ | 116.0471 | 1.9 | 1.1 | 1.3 | 1.6E-05 | 2.3E-05 | Shared |
| Acylcarnitine 10:3 | ESI+ | 309.1929 | 3.7 | 11.5 | 0.7 | 2.0E-04 | 1.7E-04 | Shared |
| Lithocholic acid | ESI- | 376.3003 | 6.8 | 8.5 | 2.0 | 2.7E-04 | 1.5E-03 | Shared |
| Hydroxyadipic acid | ESI+ | 162.0522 | 3.7 | 1.0 | 1.4 | 5.7E-04 | 6.2E-03 | Shared |
| Methylglutarylcarnitine | ESI+ | 289.1517 | 2.8 | 7.6 | 0.8 | 1.2E-03 | 9.1E-04 | Shared |
| Valyl-valine | ESI+ | 216.1468 | 2.7 | 1.9 | 0.5 | 2.7E-03 | 2.4E-03 | Shared |
| Dihydrouracil | ESI+ | 114.0430 | 0.6 | 2.7 | 1.8 | 3.8E-03 | 2.4E-03 | Shared |
| Acylcarnitine 4:0 | ESI+ | 231.1463 | 3.1 | 7.7 | 0.8 | 2.0E-02 | 9.5E-03 | Shared |
| Acetamidopropanal | ESI+ | 115.0631 | 2.3 | 2.0 | 1.5 | 2.6E-02 | 1.2E-02 | Shared |
| 3-Methyluridine | ESI- | 258.0852 | 0.1 | 1.8 | 5.4 | 2.8E-19 | 1.4E-17 | XCMS&CD |
| Pyroglutamic acid | ES+ - | 129.0423 | 2.7 | 1.8 | 2.4 | 4.0E-19 | 1.9E-17 | XCMS&CD |
| Phenylalanine | ESI+ - | 165.0784 | 3.4 | 3.8 | 1.1 | 1.7E-15 | 1.6E-10 | XCMS&CD |
| Glutamate | ESI+ - | 147.0530 | 1.5 | 1.0 | 1.6 | 3.5E-14 | 1.5E-13 | XCMS&CD |
| Homovanillic acid | ESI- | 182.0570 | 5.1 | 5.7 | 1.4 | 9.7E-14 | 3.8E-13 | XCMS&CD |
| Aconitic acid | ESI- | 174.0161 | 2.2 | 0.9 | 1.3 | 1.1E-13 | 4.0E-13 | XCMS&CD |
| Galactosylglycerol | ESI- | 254.1001 | 0.4 | 0.9 | 5.2 | 2.1E-10 | 5.4E-10 | XCMS&CD |
| Acetyl-histidine | ESI- | 197.0796 | 2.0 | 0.9 | 0.8 | 4.2E-10 | 6.5E-09 | XCMS&CD |
| N-Acetylmethionine | ESI- | 191.0609 | 3.8 | 6.3 | 1.1 | 3.6E-09 | 7.7E-09 | XCMS&CD |
| Methyladenosine | ESI+ | 281.1123 | 0.5 | 4.1 | 0.9 | 2.6E-08 | 5.3E-08 | XCMS&CD |
| N-Acetylaspartyl-  glutamic acid | ESI+ - | 304.0909 | 0.9 | 1.8 | 1.5 | 5.3E-08 | 1.1E-07 | XCMS&CD |
| 2-Ketobutyric acid | ESI+ | 102.0317 | 0.1 | 1.2 | 1.7 | 3.6E-06 | 5.8E-06 | XCMS&CD |
| 2-Pyrrolidinone | ESI+ | 85.0530 | 2.6 | 3.2 | 1.2 | 1.8E-04 | 3.3E-04 | XCMS&CD |
| Pantothenic acid | ESI- | 219.1100 | 3.2 | 5.1 | 0.9 | 4.7E-03 | 5.4E-03 | XCMS&CD |
| 5-Hydroxyindoleacetate | ESI+ | 177.0786 | 2.0 | 6.6 | 0.6 | 6.8E-03 | 7.2E-03 | XCMS&CD |
| Creatinine | ESI+ | 113.0588 | 1.3 | 1.0 | 1.1 | 3.6E-02 | 2.0E-02 | XCMS&CD |
| Undecanedioic acid | ESI- | 216.1360 | 0.9 | 7.4 | 0.6 | 1.2E-25 | 1.4E-23 | SIEVE&CD |
| Deoxyribose-phosphate | ESI- | 214.0242 | 0.2 | 0.8 | 2.3 | 3.1E-25 | 1.3E-22 | SIEVE&CD |
| Orotic acid | ESI- | 156.0180 | 5.9 | 0.8 | 4.0 | 1.2E-18 | 3.2E-17 | SIEVE&CD |
| Imidazole pyruvate | ESI- | 154.0379 | 0.5 | 4.1 | 0.2 | 1.1E-12 | 6.2E-12 | SIEVE&CD |
| Hexadecanedioic acid | ESI- | 286.2148 | 1.3 | 8.0 | 0.7 | 5.3E-12 | 1.1E-08 | SIEVE&CD |
| Valproylglycine | ESI- | 201.1362 | 1.6 | 7.0 | 0.6 | 5.7E-11 | 4.0E-10 | SIEVE&CD |
| Aspartyl-Threonine | ESI+ - | 234.0851 | 0.5 | 0.9 | 0.4 | 1.2E-10 | 4.3E-10 | SIEVE&CD |
| Acetylglutamic acid | ESI+ - | 189.0630 | 3.5 | 1.0 | 0.6 | 6.8E-09 | 1.7E-05 | SIEVE&CD |
| Heptanoylglycine | ESI- | 187.1201 | 3.8 | 6.7 | 0.6 | 1.0E-08 | 1.1E-07 | SIEVE&CD |
| FAHFA 20:2 | ESI+ | 338.2449 | 2.4 | 13.3 | 1.1 | 8.2E-07 | 1.2E-06 | SIEVE&CD |
| Acetyl-aminoadipate | ESI- | 203.0788 | 2.7 | 1.7 | 0.6 | 5.0E-06 | 8.2E-04 | SIEVE&CD |
| Acylcarnitine 8:1 | ESI+ | 285.1935 | 1.8 | 10.9 | 0.7 | 9.7E-04 | 6.9E-04 | SIEVE&CD |
| FFA 18:3 | ESI+ | 278.2239 | 2.3 | 12.8 | 1.5 | 3.8E-03 | 2.4E-03 | SIEVE&CD |
| Dihydroxypseudooxy-nicotine | ESI- | 210.1018 | 6.6 | 7.2 | 0.4 | 4.3E-03 | 3.1E-03 | SIEVE&CD |
| Tyramine-O-sulfate | ESI+ - | 217.0402 | 3.4 | 2.3 | 0.2 | 5.9E-03 | 5.4E-03 | SIEVE&CD |
| FFA 18:1 | ESI- | 282.2561 | 0.7 | 8.4 | 0.3 | 8.0E-03 | 4.4E-03 | SIEVE&CD |
| FAHFA 18:0 | ESI+ - | 314.2450 | 2.4 | 12.9 | 1.5 | 1.0E-02 | 1.1E-02 | SIEVE&CD |
| Acylcarnitine 14:0 | ESI+ | 371.3027 | 2.2 | 13.3 | 0.8 | 1.8E-02 | 8.4E-03 | SIEVE&CD |
| FFA 18:2 | ESI+ | 280.2395 | 2.5 | 13.2 | 1.1 | 1.9E-02 | 9.0E-03 | SIEVE&CD |
| O-Acetylneuraminic acid | ESI- | 309.1066 | 1.8 | 1.2 | 2.6 | 1.7E-15 | 9.4E-15 | SIEVE&XCMS |
| FAHFA 20:1 | ESI- | 340.2621 | 2.2 | 8.4 | 1.1 | 5.6E-15 | 5.4E-14 | SIEVE&XCMS |
| 2-Methylguanosine | ESI- | 297.1079 | 1.9 | 5.4 | 1.1 | 6.3E-15 | 5.9E-14 | SIEVE&XCMS |
| Succinoadenosine | ESI- | 383.1086 | 2.2 | 5.9 | 0.9 | 1.5E-14 | 1.3E-13 | SIEVE&XCMS |
| 3-Hydroxytetra  decanedioic acid | ESI- | 274.1789 | 3.3 | 7.4 | 1.1 | 1.8E-14 | 1.4E-13 | SIEVE&XCMS |
| Butyrylglycine/  Isobutyrylglycin | ESI+ | 145.0730 | 6.2 | 10.2 | 6.7 | 1.9E-14 | 8.5E-14 | SIEVE&XCMS |
| Citric acid | ESI- | 192.0267 | 1.5 | 0.8 | 1.6 | 2.8E-14 | 1.2E-13 | SIEVE&XCMS |
| Vinylacetylglycine | ESI- | 143.0575 | 4.8 | 3.9 | 1.6 | 4.0E-14 | 1.7E-13 | SIEVE&XCMS |
| Phenylpyruvic acid | ESI- | 164.0471 | 1.4 | 5.7 | 0.6 | 6.9E-10 | 2.0E-09 | SIEVE&XCMS |
| cAMP/cdGMP | ESI- | 329.0531 | 1.8 | 2.9 | 1.2 | 5.0E-09 | 1.1E-08 | SIEVE&XCMS |
| 3-Sulfopyruvic acid | ESI+ | 167.9722 | 3.7 | 14.2 | 0.6 | 5.1E-09 | 1.1E-08 | SIEVE&XCMS |
| Phenylacetylglycine | ESI- | 193.0736 | 1.5 | 6.8 | 1.3 | 2.2E-08 | 4.7E-08 | SIEVE&XCMS |
| Acetyl-glucose | ESI- | 222.0739 | 0.4 | 0.9 | 2.1 | 5.8E-08 | 1.2E-07 | SIEVE&XCMS |
| Glu-leu/Ile | ESI+ | 260.1363 | 3.6 | 7.6 | 0.9 | 4.3E-04 | 1.5E-03 | SIEVE&XCMS |
| Decanoylglycine | ESI- | 229.1675 | 1.5 | 7.3 | 0.5 | 3.7E-21 | 2.7E-19 | CD |
| Hydroxynonanoic acid | ESI- | 174.1246 | 5.5 | 7.2 | 0.6 | 4.1E-17 | 6.5E-16 | CD |
| Hydroxy palmitic acid | ESI- | 272.2353 | 0.6 | 8.1 | 0.6 | 3.6E-16 | 2.3E-15 | CD |
| Nonanoylglycine | ESI- | 215.1512 | 4.2 | 7.2 | 0.5 | 1.0E-15 | 2.1E-14 | CD |
| Methionine sulfoxide | ESI+ | 165.0458 | 1.4 | 1.2 | 1.8 | 6.2E-11 | 2.4E-10 | CD |
| Hydroxycapric acid | ESI- | 188.1404 | 4.0 | 7.5 | 0.7 | 8.9E-11 | 2.8E-10 | CD |
| Azelaic acid | ESI- | 188.1040 | 4.5 | 7.1 | 0.6 | 1.3E-10 | 5.2E-10 | CD |
| Tryptophan | ESI+ - | 204.0892 | 3.4 | 5.9 | 0.6 | 2.2E-10 | 2.4E-10 | CD |
| 2-Deoxyribonic acid | ESI- | 150.0520 | 5.8 | 0.8 | 1.4 | 1.1E-09 | 3.4E-09 | CD |
| Hexanoylglycine | ESI- | 173.1042 | 5.9 | 6.6 | 0.7 | 1.5E-09 | 1.3E-09 | CD |
| Adenine | ESI+ | 135.0544 | 1.0 | 1.1 | 0.7 | 2.3E-09 | 4.4E-09 | CD |
| Octanoylglucuronide | ESI+ | 320.1493 | 6.4 | 13.0 | 0.4 | 4.4E-09 | 9.5E-09 | CD |
| Dihydroorotic acid | ESI- | 158.0334 | 4.0 | 0.8 | 1.5 | 2.8E-07 | 2.4E-06 | CD |
| Phosphodimethy-lethanolamine | ESI+ | 169.0498 | 1.4 | 12.8 | 0.5 | 1.0E-06 | 3.0E-06 | CD |
| Hydantoin-propionic acid | ESI- | 172.0492 | 4.5 | 0.8 | 3.8 | 1.2E-06 | 2.4E-05 | CD |
| Homocitric acid | ESI- | 206.0419 | 4.0 | 1.3 | 0.7 | 3.5E-05 | 4.3E-05 | CD |
| Aminoactanoic acid | ESI+ | 159.1258 | 0.8 | 2.2 | 0.8 | 4.2E-05 | 6.0E-05 | CD |
| Threonic acid | ESI- | 136.0371 | 0.4 | 1.2 | 0.8 | 1.7E-04 | 3.1E-04 | CD |
| S-Acetyldihydrolipoamide | ESI- | 249.0871 | 5.5 | 10.2 | 0.8 | 3.1E-04 | 5.3E-04 | CD |
| Estradiol | ESI+ | 272.1771 | 2.0 | 13.4 | 0.7 | 2.0E-03 | 2.6E-03 | CD |
| Sphingosine C16:1 | ESI+ | 271.2506 | 2.0 | 13.0 | 0.8 | 3.2E-03 | 3.9E-03 | CD |
| FFA 14:1 | ESI+ | 226.1929 | 1.8 | 12.7 | 0.8 | 2.8E-02 | 2.1E-02 | CD |
| Tetradecanedioic acid | ESI- | 258.1836 | 2.0 | 7.8 | 0.6 | 5.5E-27 | 1.7E-24 | SIEVE |
| Hydroxy-undecenoic acid | ESI- | 200.1411 | 0.6 | 7.5 | 0.5 | 1.6E-17 | 3.4E-16 | SIEVE |
| 4-Keto pentadecanoic acid | ESI- | 256.2043 | 2.0 | 7.8 | 0.4 | 3.1E-17 | 5.7E-16 | SIEVE |
| Myristic acid | ESI- | 228.2094 | 2.2 | 8.2 | 0.3 | 4.3E-17 | 7.2E-16 | SIEVE |
| FAHFA 19:2 | ESI- | 324.2284 | 5.0 | 8.3 | 0.3 | 9.5E-16 | 1.2E-14 | SIEVE |
| Methylthreonine | ESI+ | 133.0733 | 4.7 | 1.8 | 2.6 | 3.6E-15 | 3.8E-14 | SIEVE |
| Acylcarnitine 2:0 | ESI- | 203.1156 | 0.7 | 6.7 | 0.6 | 1.7E-14 | 1.4E-13 | SIEVE |
| Glycerophosphocholine | ESI- | 257.1017 | 4.3 | 1.5 | 0.6 | 1.6E-13 | 1.1E-12 | SIEVE |
| FFA 15:0 | ESI- | 242.2247 | 0.6 | 8.3 | 0.3 | 2.5E-13 | 1.6E-12 | SIEVE |
| Glucono-lactone phosphate | ESI- | 258.0123 | 6.9 | 7.3 | 0.5 | 8.0E-13 | 4.6E-12 | SIEVE |
| FAHFA 24:0 | ESI- | 398.3400 | 1.0 | 9.0 | 0.5 | 4.4E-12 | 2.1E-11 | SIEVE |
| 3-Hexenedioic acid | ESI- | 144.0416 | 4.4 | 6.0 | 2.1 | 4.4E-12 | 2.1E-11 | SIEVE |
| FAHFA 18:1 | ESI- | 312.2308 | 2.5 | 8.1 | 0.7 | 1.7E-10 | 5.8E-10 | SIEVE |
| FAHFA 26:0 | ESI- | 426.3717 | 1.9 | 9.2 | 0.5 | 1.3E-09 | 3.5E-09 | SIEVE |
| Glutaric acid | ESI- | 132.0415 | 5.9 | 8.0 | 2.3 | 8.6E-09 | 1.9E-08 | SIEVE |
| FAHFA 18:4 | ESI- | 306.1839 | 2.7 | 8.2 | 0.6 | 2.2E-07 | 3.5E-07 | SIEVE |
| FAHFA 18:2 | ESI- | 310.2144 | 0.0 | 7.9 | 0.6 | 1.2E-05 | 1.3E-05 | SIEVE |
| FFA 15:1 | ESI+ | 240.2080 | 4.0 | 13.1 | 0.6 | 1.2E-04 | 1.1E-04 | SIEVE |
| Desaminotyrosine | ESI- | 166.0625 | 2.9 | 6.8 | 0.9 | 7.5E-04 | 5.5E-04 | SIEVE |
| FFA 17:1 | ESI+ | 268.2393 | 3.6 | 13.1 | 0.8 | 1.4E-02 | 7.0E-03 | SIEVE |
| 2-Aminomuconic acid | ESI- | 157.0368 | 4.5 | 3.5 | 2.4 | 2.7E-20 | 5.1E-19 | XCMS |
| Pipecolic acid | ESI- | 129.0801 | 8.5 | 1.2 | 2.2 | 9.4E-20 | 1.5E-18 | XCMS |
| Homocysteinesulfinic acid | ESI- | 167.0239 | 7.8 | 1.0 | 2.3 | 4.1E-15 | 2.1E-14 | XCMS |
| Tryptamine | ESI+ - | 160.1006 | 3.7 | 7.2 | 2.4 | 2.0E-14 | 8.9E-14 | XCMS |
| Dihydroxybutan phosphate | ESI- | 184.0153 | 8.7 | 0.8 | 3.8 | 4.4E-14 | 1.8E-13 | XCMS |
| Glucosyl-glycerol phosphate | ESI- | 334.0687 | 6.6 | 1.5 | 3.7 | 1.3E-12 | 4.2E-12 | XCMS |
| 2-(4-Hydroxybenzyl) isoindole-1,3-dione | ESI- | 253.0726 | 5.1 | 0.9 | 8.1 | 1.6E-12 | 5.2E-12 | XCMS |
| Hydroxydiphenylamine | ESI- | 185.0844 | 1.8 | 1.3 | 1.9 | 5.6E-12 | 1.7E-11 | XCMS |
| Tyrosine methylester | ESI- | 195.0904 | 4.4 | 7.7 | 1.7 | 5.8E-10 | 1.4E-09 | XCMS |
| Acetyl-acetylneuraminic acid | ESI- | 351.1155 | 3.0 | 1.4 | 1.8 | 3.7E-09 | 8.3E-09 | XCMS |
| Butylpiperidine | ESI+ | 141.1511 | 4.6 | 12.5 | 0.8 | 5.2E-06 | 8.1E-06 | XCMS |
| Uridine diphosphate glucose | ESI- | 566.0556 | 1.0 | 7.5 | 2.4 | 5.0E-05 | 6.2E-05 | XCMS |
| Cysteinylglycine | ESI+ | 178.0404 | 4.6 | 0.8 | 0.9 | 2.0E-04 | 2.2E-04 | XCMS |
| Dimethyllysine | ESI+ | 174.1356 | 7.1 | 12.2 | 1.4 | 7.4E-04 | 6.3E-03 | XCMS |
| FAHFA 23:6 | ESI- | 372.2308 | 2.0 | 8.1 | 2.0 | 1.5E-03 | 1.0E-03 | XCMS |
| 2-Oleoylglycerophospho-choline | ESI+ | 522.3556 | 0.7 | 13.9 | 1.7 | 1.7E-03 | 1.6E-03 | XCMS |
| FAHFA 26:1 | ESI+ | 424.3589 | 8.6 | 13.2 | 0.7 | 1.3E-02 | 4.3E-03 | XCMS |
| FFA 19:0 | ESI+ | 296.2706 | 3.1 | 13.5 | 0.8 | 4.8E-02 | 3.3E-02 | XCMS |

**Supplemental Table S2**. Clinical information of recruited human subjects.

|  | Control | Preclinical AD | Total |
| --- | --- | --- | --- |
| Sample size | 14 | 16 | 30 |
| Male/Female | 7/7 | 6/10 | 13/17 |
| Age | 60±7 | 61±6 | 61±6 |

**Supplemental Figure S1**. Custom workflow in Compound Discoverer software. Post-processing node is differential analysis with *t*-test (not illustrated in the figure).
